# Preliminary Report on a Method of Fluoroscopic Examination with the Army Bedside Unit

**Published:** 1919

**Authors:** 


***Preliminary Report on a Method of Fluoroscopic Examination with the Army Bedside Unit.*** By Captain FRANCIS G. BORZELL.

During the last 3 months over 150 cases have been examined at the bedside in Base Hospital No. 38. The examinations have been both radiographic, with and without the intensifying screen, and fluoroscopic, for fractures, foreign bodies and pulmonary conditions of those patients who could not be transported to the laboratory.

The following method of examination was used with success in a number of cases requiring radiologic studies to determine the presence of fluid, empyema, hemothorax, pneumonia, pericardial effusion, etc. Many of the patients were either too sick to be moved or else immobilized in Balkan frames and other devices.

The bed was raised about 12 inches, by placing 4 stilts under the legs, the tube was dropped beneath it, and very satisfactory information secured by fluoroscoping through the mattress. The bed springs of the regulation bed do not interfere materially with the study, owing to the fact that being at a greater distance from the screen than the tissues to be examined, the mesh shadow is very much exaggerated and but a few of the lines of the spring intercept vision. The average ward bed spring casts a shadow which places the spring shadow 20 cm. apart on the screen. The Balkan frame, when used, is lashed to the bed and lifted with it, resting with the legs of the bed on the stilts.

In hospitals of the barrack type, the bedside unit is suspended by the handles on a litter made of two timbers fastened together by two cross pieces and thus carried from ward to ward.

A bedside examination by this method affords :

1. Easy access to entire torso and limbs, fluoroscopically.
2. Patients can be examined who would otherwise be unavailable.
3. No risk to very sick pulmonary cases.

